# Chemical and sensory properties of young cabernet sauvignon and marselan wines from subregions on the eastern foothills of helan mountains in ningxia, China: Terroir effect

**DOI:** 10.1016/j.fochx.2025.102191

**Published:** 2025-01-21

**Authors:** Xue Zhang, Hui Yang, Na Liu, Jian Sun, Ruijia Yao, Fangzhou Shi, Jiming Li, Wenguang Jiang, Hongying Li, Qingchen Zhang, JunXiang Zhang

**Affiliations:** a School of Life Sciences, Ningxia University, Yinchuan, Ningxia 750021, China; b School of Wine & Horticulture, Ningxia University, Yinchuan, Ningxia 750021, China; c Institute of Medical Sciences, Ningxia Medical University, Yinchuan 750004, China; d Ningxia Changyu Longyu Estate Co. Ltd., Yinchuan, Ningxia 750000, China; e School of Advance Interdisciplinary, Ningxia University, Zhongwei, Ningxia 750021, China; f Ningxia Institute of Meteorological Sciences, Yinchuan, Ningxia 750002, China; g College of Pharmacy, University of Florida, Gainesville, FL 32610, USA

**Keywords:** Geographical indication, Wines, Terroir, Volatile compounds, Phenolics

## Abstract

This study aimed to analyze the characteristics of Cabernet Sauvignon (CS) and Marselan (M) wines from different subregions on the eastern foothills of Helan Mountain. UHPLC–ESI–Q–ToF and HS–SPME–GC–MS were employed to analyze the metabolic properties of the wines, and QDA was combined for sensory characterization. The results indicated that chromaticity, total phenols, ethyl isobutyrate, n-decanoic acid, (−)-epigallocatechin, and epigallocatechin were key indicators for distinguishing CS wines from different subregions, whereas total acids, total phenols, hexanol, ethyl butyrate, protocatechuic acid, and (+)-catenin were key indicators for distinguishing M wines from different subregions. The richness and coordination of fruit, floral, dried fruit, spice, and green flavors in the wine were key indicators determining the flavor characteristics of wine in winemaking area. The key compounds with aroma of green, fruity, and floral that determine the core aroma, aroma coordination, and elegance of wine in the winemaking area include cis-2-exen-1-ol, ethyl palmate, octanoic acid, and n-decanoic acid.

## Highlights

• The richness and coordination of fruit, floral, dried fruit, spice, and green flavors in the wine were key indicators determining the flavor characteristics of wine in winemaking area.
• The key compounds that determine the core aroma, aroma coordination, and elegance of EFHM wine were cis-2-exen-1-ol, ethyl palmitate, octanoic acid, and n-decanoic acid.
• EGC and EC, PA and C, are the key phenolic compounds used to distinguish regions in Cabernet Sauvignon and M wine, respectively.
• Ethyl isobutyl and n-decanoic acid, hexanol and ethyl butyl, are key volatile compounds used for region identification in CS and M wines, respectively.
• Wines produced under conditions of lower accumulated temperature, longer sunshine hours, and sandy or yellow loam soils have better quality.

## 1 Introduction

**Fig. 1:**
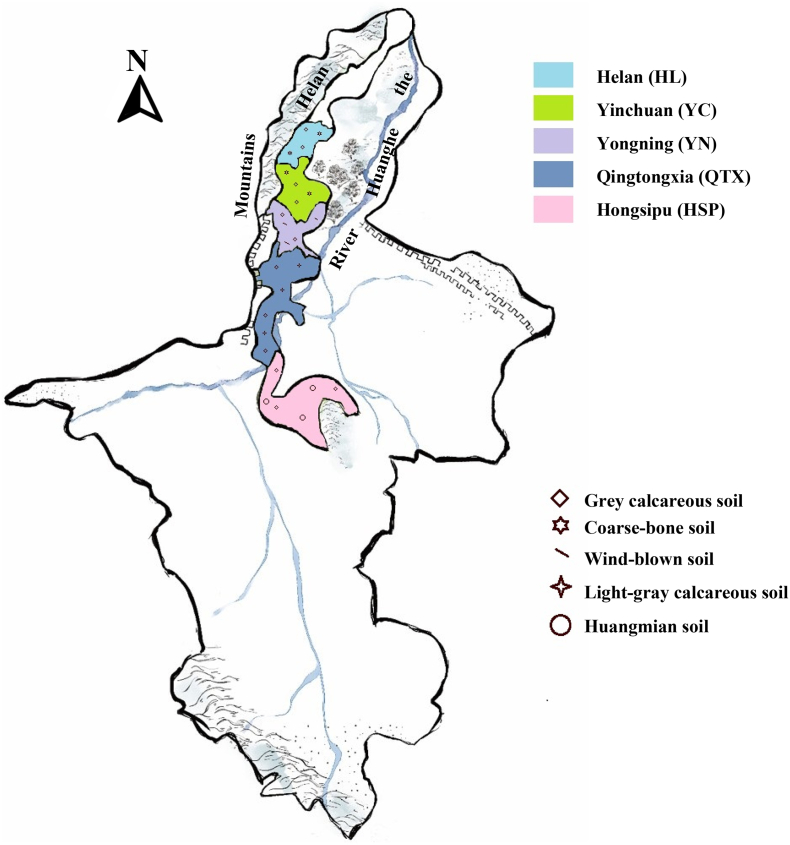
**Region Map of the EFHM in Ningxia.** The background image of this picture is quoted from https://baijiahao.baidu.com/s?id = 1,710,866,027,442,930,742&wfr = spider&for = pc.

The eastern foothills of the Helan Mountain (EFHM, 37°43′-39°23′N, 105°45′-106°47′E) is a vast area between the Helan Mountain alluvial fan and the Yellow River alluvial plain, known for its proximity to Helan Mountain to the west (Fig. 1). With its unique natural environment and increasingly mature grape (*Vitis vinifera*) cultivation techniques, it has become an emerging high-quality wine region in China. The identification of famous wines, such as Château d'Yquem, Champagne, and Romanée-Conti, is based on their geographical location, which involves the concept of origin. Wines from designated geographical regions are usually considered to have a unique style endowed by the terroir(Green, Parr, Breitmeyer, Valentin, & Sherlock, 2011). Thus, EFHM wine identification is an essential and urgent step toward brand maturity. Red wines from this region exhibit diverse flavor characteristics, mainly due to differences in geographical location, climatic conditions, and soil composition between different subregions. These factors can affect the quality of wine by influencing the growth and development of grapevines, including the accumulation of metabolites such as phenols and aromas. Research on the influence of terroir on phenolic compounds has shown that high temperature, light, water scarcity, and large temperature differences between day and night can upregulate gene expression related to flavonoid metabolism and increase flavonoid synthesis. However, in the hottest month (during berry ripening), a certain range of low temperatures (such as in high-altitude areas) are beneficial for the accumulation of phenolics, such as flavonols, flavanols, and hydroxycinnamic acid, whereas excessively high temperatures can negatively affect their accumulation(Robinson et al., 2011; Wei et al., 2023). Soil type and nutrients can affect the accumulation of anthocyanins. For example, sandy soil and soil rich in phosphorus, calcium, and potassium, which contribute to the accumulation of anthocyanins and produce better color in wine(Chen, Yang, Deng, Lei, & Xu, 2020; Lasa et al., 2012; Tian et al., 2023). Research on the influence of terroir on aroma compounds has found that light, temperature differences, and soil types can affect the accumulation of isoprene (Friedel et al., 2016), methoxypyrazine (Mendez-Costabel et al., 2014), volatile thiols(Des Gachons et al., 2005), C6 alcohols(Lu et al., 2023), and aroma precursors, such as fatty and amino acids in wine(Waterhouse, Sacks, & Jeffery, 2016). The accelerated degradation of carotenoids in grape fruits under strong light exposure increases the synthesis of isoprene (Robinson et al., 2011). Wines produced in vineyards with higher soil pH have lower C6 alcohols and herbal aromas (Lu et al., 2023), whereas wines made from grapes grown on sandy soil have better aromas(Wang, Sun, & Chang, 2015). The terroir of different geographical locations has diversity; therefore, the resulting wines often have unique regional characteristics.

Cabernet Sauvignon (CS) has become the main variety in the EFHM region owing to its excellent brewing potential and strong adaptability. The produced wine has a strong fruity, spice, and green flavor as well as a complex and full body(Ayestarán et al., 2019). Marselan (M) has also become a popular wine variety in the EFHM region owing to its feature of drought and disease resistance. The produced wine has a strong fruity and herbal aroma(Sun, Zhang, Xia, Zhang, & Zhang, 2023).

In recent years, with the deepening of research on wine flavor chemistry, many studies on the regionalization of wine terroir have emerged one after another(Duley et al., 2021; Lola et al., 2023). The study of wine flavor in EFHM regions has also gained considerable attention and involved research on phenols, aroma, and sensory aspects(Song et al., 2023; Zhang, Wang, Zhang, Zhou, & Yuan, 2024; Zhang et al., 2021; Zhao et al., 2023). However, the study is generally limited to the overall performance of the region or the analysis of certain substances. Regarding terroir, it is only related to temperature and precipitation, and there is still a lack of comprehensive research on the flavor and chemical performance of the main varieties in the region under the influence of terroir conditions in different subregions. Thus, to reduce the influence of factors other than terroir, this study focused on the unaged CS and M young wines from five representative regions in 2021 and 2022. Solid-phase headspace microextraction combined with gas chromatography–mass spectrometry (HS–SPME–GC–MS) technology was used for the qualitative and quantitative analyses of volatile compounds. Ultrahigh-performance liquid chromatography quadruple time-of-flight mass spectrometry technology (UHPLC–ESI–Q–ToF) was used for the qualitative and quantitative analyses of monomeric phenolic compounds, and quantitative descriptive analysis (QDA) was employed to characterize the sensory characteristics of the wine, aiming to elucidate the chemical flavor and sensory characteristics of wines from different winemaking areas in the EFHM, Ningxia. Furthermore, using correlation analysis, the compounds that affect flavor characteristics were explored to better elucidate the main chemical components that affect the flavor characteristics of EFHM wine. By leveraging environmental factors to endow wine with chemical memory, fingerprint maps of CS and M wines from the different subregions of EFHM were constructed, which can provide data support for the analysis of the flavor characteristics of wines from different subregions and to better understand the potential for flavor development of wines.

## 2 Materials and methods

### 2.1 Selection of wine samples

A total of 36 samples of CS and M wines were collected for the experiment, all of which were bottled without aging after malolactic fermentation in 2021 and 2022. Samples were all sourced from five representative regions, divided by geographical location from south to north, namely Hongsibao (HSP), Qingtongxia (QTX), Yongning (YN), Yinchuan (YC), and Helan (HL) production regions(Fig. 1). There are differences in the climate and soil types among the different subregions. Due to the decreasing altitude of the Ningxia Plain from south to north and from west to east, the temperature in the northern regions is generally higher than that in the southern regions, and the annual precipitation gradually decreases from south to north. Thus, the HSP region in the southernmost part has the lowest accumulated temperature, highest precipitation, and longer sunshine hours, whereas the HL and YC regions in the north have higher accumulated temperature, lower precipitation, and shorter sunshine hours (J. Wang et al., 2020). From the perspective of soil types, the HSP region is mainly composed of loess soil, the YN region is mainly composed of sandy and irrigated soils, and the remaining regions are mainly composed of calcareous soil(R. Wang et al., 2015). For detailed information, see Supplementary Table S1.

### 2.2 Chemicals and standards

Reagents: The total phenol detection kit and anthocyanin detection kit were purchased from Biosystems, Spain. Anhydrous ethanol (chromatographically pure) was purchased from Shanghai Aladdin Reagent Co., Ltd. Hydrochloric acid was purchased from Tianjin Miou Reagent Co., Ltd., Tianjin, China, and the monomeric phenolic substance standard (chromatographic purity) was obtained from the National Institutes of Food and Drug Control, Beijing, China. In addition, formic acid, methanol, acetonitrile (chromatographic purity), and ethanol (chromatographic grade) were obtained from Fisher Corporation, USA. NaCl was purchased from Sinopharm Chemical Reagent Co., Ltd., Shanghai, China.

Standards: A total of 65 volatile compound standards (see Supplementary Table S3), including ethyl acetate, ethyl isobutyrate, and ethyl butyrate, were obtained from Sigma-Aldrich, St. Louis, MO, with purities of over 90 %, whereas monomeric phenol standards (Table S3) were obtained from the National Institutes for Food and Drug Control, Beijing, China, with a purity of over 98 % (see Supplementary Table S4).

### 2.3 Oenological parameter determination

Residual sugar, total acid, alcohol content, and sulfur content are determined by referring to the corresponding direct titration, indicator, distillation, density bottle, and direct iodometric methods in GB/T 15038–2006 “General Methods for Analysis of Wine and Fruit Wine” (Committee, 2008). The pH was measured using a Thunderbolt PHS-3C pH meter (Shanghai, China). The total tannins were determined using the Bate–Smith method(Bate-Smith, 1973), and the chromaticity tone was determined using the method of Glories et al.(Glories, 1984). The total phenols and total anthocyanins were determined using a Y15 wine automatic analyzer (Guangzhou, China). The experimental principle of the total phenols was based on the reaction between polyphenols in the sample and phenol reagent in an alkaline environment, and the increase in color in the sample was proportional to the concentration of polyphenols. The experimental principle of total anthocyanins was based on the absorbance of the sample at 520 nm being proportional to the concentration of anthocyanins in a certain environment. This method determines the ionized and ionizable anthocyanins present in the sample.

### 2.4 Volatile compound analysis via HS–SPME–GC–MS

The analysis of volatile compounds adopted the methods established in the laboratory in the early stage (Z. Zhang et al., 2023). The volatile extract was desorbed at 250 °C for 10 min without splitting. The oven temperature for the DB-Wax column (60 m × 0.25 mm id × 0.25 μm; J&W Scientific, Folsom, CA, USA) was initially held at 50 °C for 1 min, elevated to 220 °C at 3 °C/min, and maintained for 5 min. The temperature of the transfer line was set to 250 °C. The electron ionization source was used, with a source temperature of 230 °C and an electron energy of 70 eV, and the scan mode of the MS detector was 29–350 *m*/*z*. The volatiles were identified using the NIST 17 spectral library and verified using the linear retention indices of C8–C20 alkanes (Sigma-Aldrich, Shanghai, China) on a DB-Wax column. 4-Methyl-2-pentanol was used as an internal standard. The 65 standard samples were diluted at a ratio of 1:2 to prepare 10 gradient standard stock solutions to obtain standard curves for quantification.

### 2.5 Phenolic compound qualitative analysis via UHPLC–ESI–Q–ToF and quantitative analysis via HPLC–DAD

Monomeric phenols were detected using the method of Liang et al.(Liang, Huang, Zhang, & Fang, 2023) with slight modifications.

Qualitative Analysis. The chromatographic column was Agilent ZORBAX Eclipse Plus C18 (4.6 × 250 mm 5 μm). The mobile phases were water (A) and methanol (B), both containing 0.1 % formic acid; the injection volume was 10 μL, and the column temperature was 30 °C. The elution program was performed at a flow rate of 1.0 mL/min: 5 % B (1 min), 5 %–16 % B (1 min), 16 %–20 % B (1 min), 20 %–23 % B (7.5 min), 23 %–24 % B (4 min), 24 %–25 % B (1 min), 25 %–26 % B (2.5 min), 26 %–27 % B (8 min), 27 %–28 % B (2 min), 28 %–30 % B (4 min), 27 %–28 % B (2 min), 28 %–30 % B (4 min), 30 %–35 % B (6 min), 35 %–45 % B (11 min), 45 %–47 % B (3 min), 47 %–48 % B (5 min), 48 %–50 % B (10 min), 50 %–80 % B (1 min), 80 %–100 % B (0.5 min), 100 % B (4.5 min), and 100 %–5 % B (2 min).

Quantitative analysis. An Agilent Technologies 1220 Infinity II LC (Santa Clara, CA, USA) connected to an Agilent 1220 series DAD was used to quantify the phenolic compounds. The column and elution condition were the same as those mentioned in the qualitative analysis via HPLC–ESI–Q–TOF. Standard samples were used to draw standard curves for quantification.

### 2.6 Sensory analysis

The sensory analysis adopted QDA, which is widely used in the sensory evaluation of wine and can achieve overall rating and personalized description of sensory attributes (Cadot, Caillé, Samson, Barbeau, & Cheynier, 2010). Includes 11 common aromas, as well as overall evaluations of color, taste, and aroma (see Supplementary Fig. S3). The sensory performance were evaluated by 16 undergraduate and graduate students (4 men and 12 women, the age for drinking wine is not less than 3 years) who voluntarily participated in the grape and wine research. The aroma properties of the wines were scored, and their specific aromas were analyzed.

### 2.7 Statistical analysis

Microsoft Excel 2019 was used for basic data statistics and to draw bar and radar charts. Analysis of variance (ANOVA), hierarchical cluster analysis (HCA), principal component analysis (PCA), and correlation analysis were conducted on the agricolae, pheatmap, factoextra, and ggcorrplot packages of the R (3.6.2) statistical analysis software, respectively.

## 3 Results and discussion

### 3.1 Analysis of the oenological parameter, phenolic indices, and volatile compounds of the CS and M wines

The quality of wine largely depends on the quality of the raw materials, which are influenced by several factors, such as climate, soil, and microorganisms, in the terroir. Thus, the accumulation and evolution of physical and chemical indicators, phenols, and aroma of wine were determined at the terroir source.

The main climatic characteristics during the critical growth period of EFHM grapes were hot and rainless, high accumulated temperature, long sunshine duration, and large temperature difference between day and night (see Supplementary Fig. S1 & Table S1). Higher cumulative temperature and lower precipitation are conducive to sugar accumulation (Blttel, Pfeiffer, & Wirth, 2009), and the alcohol content of EFHM wine is generally high (14.78 %–15.73 % vol). Among them, the alcohol content of the wine samples from the HSP region was the highest (*P* < 0.05), and the color of the wine samples from the HL region was the highest (*P* < 0.05). Correlation analysis was conducted on the oenological parameters (see Supplementary Fig. S2), and significant correlations were observed between indicators, such as chromaticity, pH, volatile acid, and residual sugar, particularly between chromaticity and residual sugar. Perhaps due to the correlation between sugar concentration and color with berry maturity, particularly the expression of several enzymes involved in the phenylpropanoid pathway that affects the formation of phenolic substances, which may be controlled by sugar(Martínez-Gil, Gutiérrez-Gamboa, Garde-Cerdán, Pérez-Álvarez, & Moreno-Simunovic, 2018). Furthermore, the range of hue of the wine was 0.76–0.83, and the samples from each subregion all conformed to the characteristics of young wine (hue <1).

**Fig. 2:**
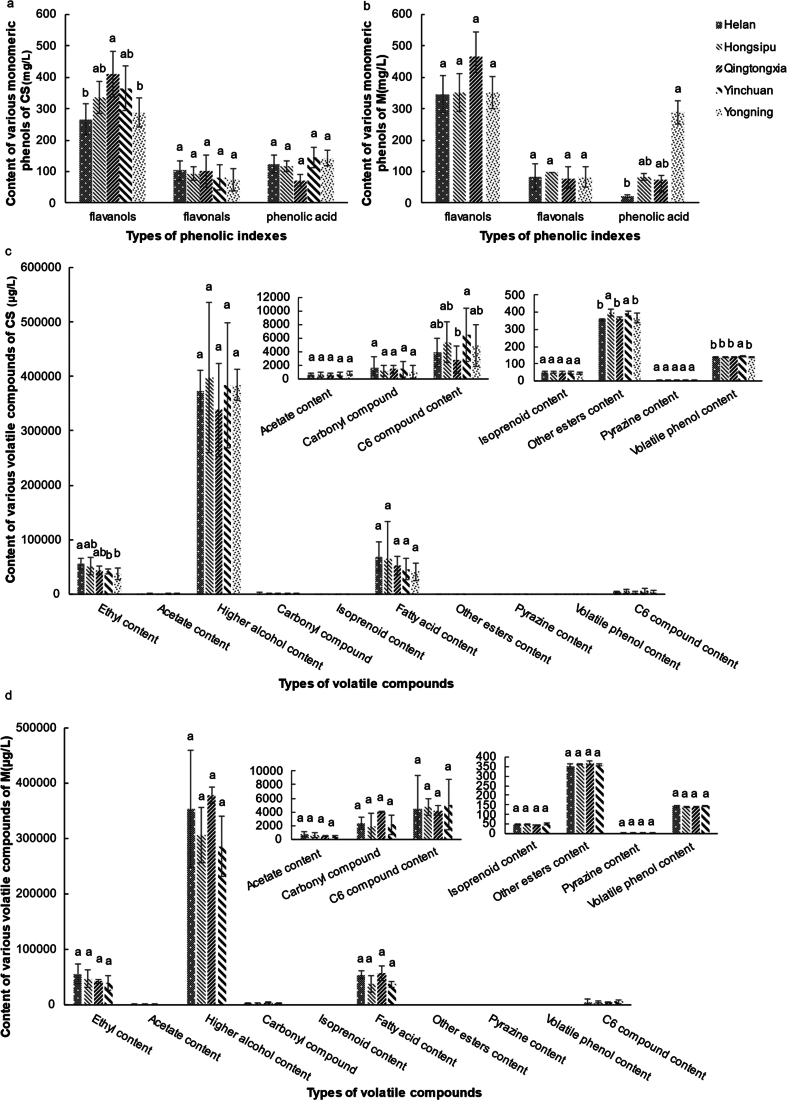
**Phenol and volatile compound contents of CS (a, c) and M (b, d) wines in the five subregions of EFHM.** Different Latin letters indicate significant differences according to the Tukey HSD test (*P* < 0.05).

The vast majority of phenolic compounds in wine originate from grape berries, with some coming from oak products used in production or winemaking processes (Waterhouse et al., 2016). Affected by various factors, such as climate, soil, and technology(Waterhouse et al., 2016), it is an important landmark in the study of terroir in regions. In this study, a total of 16 monomeric phenols were quantified, including 7 flavanols, 2 flavonols, and 7 phenolic acids (see Supplementary Fig. S4). Among them, monomers such as (+)-catechin (C), epicatechin (EC), (−)-gallocatechin (GC), (−)-epigallocatechin (EGC), (−)-epicatechin gallate (ECG), etc. of flavan-3-ol are derived from grapes.(Waterhouse et al., 2016). Flavanol monomers are the source of bitterness and astringency in wine and the most abundant phenolics in EFHM wine(Waterhouse et al., 2016)(Fig. 2a & b). The content of flavanols in wine from the QTX region is higher than those in other regions, particularly in CS wine. The high altitude, relatively low accumulated temperature, less precipitation, and large temperature difference in this region may be the key factors affecting flavanol accumulation. Wines from the YN region have high levels of phenolic acids, particularly M wines. The relatively low temperature in the hottest month (especially in 2022; see Supplementary Fig. S1) may be the reason for the high phenolic acid content in this region (Martínez‐Gil, Gutiérrez‐Gamboa, Garde‐Cerdán, Pérez‐lvarez, & Moreno-Simunovic, 2018).

Aroma is a key trace component that determines the quality of wine and an important marker in the study of terroir in winemaking regions. Owing to the growth and development of fruits, they can affect the accumulation of primary and secondary aroma precursors, ultimately impacting the production of secondary metabolites, such as esters, higher alcohols, and isoprene in wine(Waterhouse et al., 2016). Therefore, volatile compounds can, to some extent, reflect the terroir effect. In this study, the aroma of 36 young CS and M wines aged 21 and 22 were analyzed, with a focus on the aroma of the variety and the aroma of the fermentation. A total of 65 volatile compounds were qualitatively and quantitatively detected, including 12 higher alcohols, 12 ethyl esters, 5 acetate esters, 6 other esters, 6 fatty acids, 8 isoprenoids, 7 aldehydes and ketones, 3 volatile phenols, 1 pyrazine, and 6 C6 alcohols (see Supplementary Table S3). Among them, higher alcohols had the highest content, with an average content between 300 and 400 mg/L (Fig. 2c & 2d). When the content of this substance is below 300 mg/L, it will increase the complexity of the aroma, if it is above 400 mg/L, it may inhibit the release of other aromatic compounds, which will negatively affect the aroma of wine(Ayestarán et al., 2019). The content of higher alcohols in EFHM wine is relatively moderate. Esters and fatty acids are also compounds with higher content in wines. The ethyl ester content of CS wine in HL region is significantly higher than that in others. The content of other esters in CS wine from the YN, YC, and HSP regions is significantly higher than those in other regions. The contents of compounds, such as pyrazine, volatile phenols, and terpenes, in wine are relatively low. However, the odor threshold is often lower, for example, the threshold of 2-methoxy-3-isobutyl pyrazine is 0.002 μg/L, which can contribute to green odors, such as bell peppers, to the wine region(Waterhouse et al., 2016). The contents of volatile phenols and C6 compounds in CS wine from the YN and YC regions are significantly higher than those in other subregions (Fig. 2a & 2b), and the higher content of volatile phenols may be related to the higher content of precursor phenolic acids (Waterhouse et al., 2016). The differences in the accumulation of different compounds between regions are unique chemical imprints created by the terroir.

### 3.2 Sensory analysis of CS and M wines from five winemaking areas of the EFHM

Sensory analysis is an important part of wine quality identification and analysis and is key to distinguishing the characteristics of wine in the region. The aroma characteristic contour maps and scoring maps of the CS and M wines from the five subregions of EFHM are shown in Supplementary Fig. S3. Fruit, floral, green, spice, and dried fruit aromas are the five core wine aromas in the region. These core aromas of the YN wine region are outstanding, particularly fruity, green, and floral aromas. This may be due to the favorable sandy soil conditions for grape ripening in the region, which promotes the accumulation of other esters, volatile phenols, and C6 compounds, resulting in good aroma performance, higher richness, and aftertaste scores of the wine(R. Wang et al., 2015). In addition, the mushroom flavor in the winemaking area may be caused by a high proportion of yeast (*Crustomyces subabruptus*) attached to the surface of the grapes(Delcros, Collas, Hervé, Blondin, & Roland, 2023). The aroma characteristics of the CS wine from the QTX region are relatively prominent green and spice flavors, which may be due to the relatively cool climate, clayey calcareous soil, and sufficient water supply from the drip irrigation technology, resulting in the accumulation of more compounds, such as 4-ethylguaiacol (volatile phenol), cis-2-exen-1-ol, 1-octen-3-ol (C6 alcohol), 1-hetanol (higher alcohol), and (Z)-aok-lactone (ester) with green and spice flavors in the region(Leeuwen et al., 2020; Stefanos & Elements, 2018; R. Wang et al., 2015). The CS wines from this region also have an unpleasant pickle flavor, which could be the reason for the low overall score of the wine region. The CS wine from the HSP region has a prominent floral aroma, rich fruity aroma, weak green flavor, and high scores in elegance, coordination, and aftertaste. Special yellow loess soil, lower accumulated temperature, and longer sunshine hours may be beneficial for the accumulation of ethyl lactate with floral fragrance and phenols. The floral and fruity aromas enhance sweetness through the interaction of taste and aroma(Ayestarán et al., 2019), thereby increasing pleasant sensory enjoyment, which could be the reason for the good elegance and coordination of this region. The characteristic of M wine in this region is its prominent green, floral, and spice flavors. However, this could be due to the overly prominent green aroma, which disrupts the balance and reduces the pleasure of the aroma, resulting in lower scores for aroma coordination and elegance. The fruity aroma is the most prominent aroma in CS wines from the YC region; the green and spice flavors are also relatively prominent. The prominent aromas in CS wines from the HL region include green, fruity, spice, and mushroom flavors. The prominent aroma performance of the two winemaking areas is quite similar. The characteristics of high accumulated temperature, low precipitation, and short sunshine hours in the HL and YC regions may be conducive to the accumulation of esters with fruity aroma and C6 compounds with herbal aroma. The fruity, floral, and mushroom aromas were prominent in the M wines from the HL region. From the perspective of sensory scores, it was observed that wines made under lower accumulated temperatures, longer sunshine hours, and terroir with sandy or yellow loam soil obtained a more balanced aroma. The commonalities and characteristics exhibited by wines from different subregions in sensory representation are also unique imprints of terroir formation.

### 3.3 Classification of wines by geographical origin based on oenological parameters

In addition to conducting ANOVA and sensory analysis, it is usually expected to combine PCA and HCA to achieve terroir discrimination by using the differential expression of chemical parameters in the subregion interval. Among them, PCA is employed to explain most of the variations in the original data with fewer variables, extract the most important features from the data, achieve dimensionality reduction and simplification of the data, and make data processing and analysis more efficient and intuitive. Heatmap is a hierarchical clustering method that uses the degree of difference and similarity between multiple sets of values as a basis to obtain the distance relationship between clusters in the samples. It mainly represents data with colors and demonstrates the relationship between samples in a more intuitive way through virtualization(Liu et al., 2014). To achieve better discrimination results, the study sequentially analyzed the oenological parameters, volatile compounds, and phenolic compounds.

#### 3.3.1 Classification of wines by geographical origin based on the oenological parameters

**Fig. 3:**
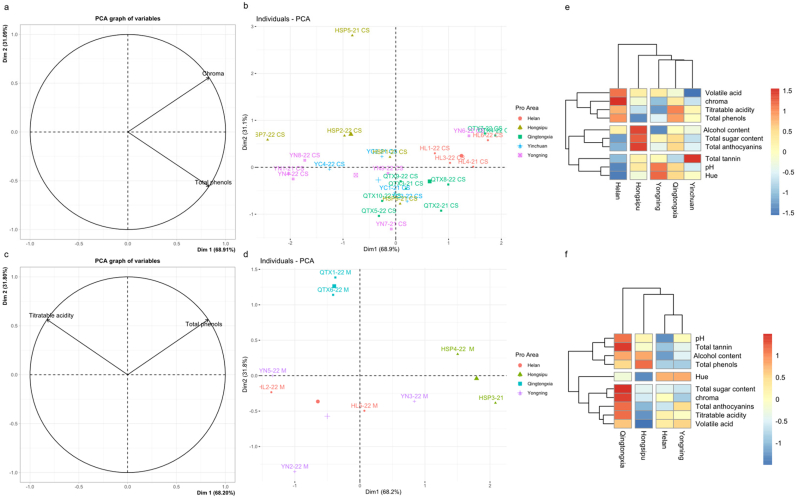
**Factor maps (a & c, *P* < 0.01), individual factor maps (b & d, *P* < 0.01), and heatmap (e & f) of CS and M wine oenological parameters in EFHM.** The horizontal axis represents the wine region; vertical axis, the oenological parameter; and blue to red, the content from low to high of e & f.

Oenological parameters are the first physical and chemical parameters that need to be monitored and tested in wine production as they reflect the quality and stability as well as the terroir characteristics of wine to some extent. First, the correlation between CS and M wines from the different subregions of EFHM and related key oenological parameters was analyzed via PCA, as shown in Fig. 3. In CS wines, the contribution rate of PC1 was 68.9 %, whereas that of PC2 was 31.1 %. In M wines, the contribution rates of PC1 and PC2 were 68.2 % and 31.8 %, respectively. Chroma and total phenols in the variable plot were the main vectors with significant differences obtained through significance screening in the CS wine (Fig. 3a), whereas titratable acidity and total phenols were the main vectors with significant differences in the M wine (Fig. 3c). The distribution of wine samples from different subregions in different quadrants was achieved through these relevant indicators with significant differences. The distribution of CS wines from different subregions is shown in Fig. 3b. PC1 coincides with wines of HL with positive coordinates and is positively correlated with total phenols and chromaticity. QTX wines showed a positive correlation with total phenols, and opposite to PC2, it is positively correlated with total phenols. Furthermore, the wines from HSP had a negative correlation with total phenols. The wines from YN and YC had a negative correlation with chroma and total phenols. The distribution of M wines from different regions is illustrated in Fig. 3d. PC1 coincided with the wines of HSP with positive coordinates and showed a positive correlation with total phenols. PC2 was consistent with the wines of QTX and showed a positive correlation with titratable acidity and total phenols. The wines from HL and YN had a negative correlation with titratable acidity and total phenols, which was opposite to PC2.

To better understand the similarities and differences of CS and M in the different winemaking regions of the EFHM, HCA was conducted using oenological parameters as variables to explore the distance of terroir relationships among wines from different subregions. The contour maps of the oenological parameters for the CS and M wines are shown in Fig. 3e and 3f. According to the similarity of oenological parameters, the parameters were divided into three groups from top to bottom, and the subregions were divided into three clusters from right to left. The contour maps of the oenological parameters of the CS wine are shown in Fig. 3e. Among them, YC, QTX, and YN, three winemaking subregions located in the middle of the region, were clustered together in the shortest hierarchical distance owing to the similar contours of their oenological parameters. This could be attributed to the similarity of terroir in these 3 closer subregions. The HSP region located at the southern end of the region and the HL region located at the northern end of the region were separately clustered. The clustering effect effectively reflected the distance of the geographical location. The higher content of the first group of substances such as volatile acids, chromaticity, and total phenols compared to other subregions is the reason for the farthest clustering distance in the HL region, which corresponds to the results in Table 1, where their chromaticity is significantly higher than those of other subregions. The key difference between the HSP regions and other subregions lies in the higher alcohol content, residual sugar, and total anthocyanin content in the second group of substances. The outline of the M wine oenological parameters is shown in Fig. 3f. The HL and YN regions were clustered together owing to their similar overall physical and chemical profiles. The HSP and QTX regions located at the southern end of the region were clustered separately. Among them, the contour of the wines in the QTX region was significantly different from those in the other subregions. Among the three clustering groups in this region, the majority of substances in the first and third groups have the highest content in several winemaking areas. The key difference between the HSP regions and other regions is that the total phenolic content in the first group of substances is high, whereas the total and volatile acid contents in the third group of substances is low. HCA intuitively presents the distance relationship between subregions through the approximation of winemaking parameter contours and is consistent with the above ANOVA and PCA results.

**Table 1:** Oenological parameters of wines from the five subregions of EFHM of Ningxia (N-S, *p* < 0.05).

	Pro Area	Helan (HL)	Yinchuan (YC)	Yongning (YN)	Qingtongxia (QTX)	Hongsipu (HSP)
Cabernet Sauvignon (CS) &Marselan (M)	Alcohol content (*v*/v, %)	15.22 ± 0.20^ab^	14.90 ± 0.49^ab^	14.78 ± 1.25^b^	15.20 ± 0.64^ab^	15.73 ± 0.34^a^
	Residual sugar (g/L)	4.00 ± 0.95^a^	3.98 ± 0.60^a^	4.40 ± 0.70^a^	4.64 ± 0.57^a^	4.77 ± 0.46^a^
	Titratable acidity (g/L, Tartaric acid)	6.13 ± 0.60^ab^	5.95 ± 0.78^ab^	5.33 ± 0.71^b^	6.44 ± 0.65^a^	5.30 ± 0.98^b^
	Volatile acid (g/L)	0.55 ± 0.04^a^	0.42 ± 0.07^a^	0.53 ± 0.08^a^	0.50 ± 0.10^a^	0.48 ± 0.12^a^
	Free SO_2_	29.5 ± 4.85^b^	35.75 ± 5.32^a^	30.67 ± 4.61^b^	36.00 ± 2.26^a^	33.43 ± 2.50^ab^
	pH	3.56 ± 0.22^b^	3.61 ± 0.18^ab^	3.82 ± 0.12^a^	3.78 ± 0.29^ab^	3.75 ± 0.17^ab^
	Total anthocyanins(mg/L)	365.00 ± 67.70^a^	374.00 ± 128.91^a^	407.89 ± 88.79^a^	431.75 ± 130.79^a^	420.71 ± 94.49^a^
	Total tannin(g/L)	3.41 ± 1.36^b^	5.50 ± 0.99^a^	4.08 ± 1.48^ab^	4.07 ± 1.07^ab^	4.27 ± 1.00^ab^
	Total phenols(mg/L)	2322.58 ± 139.76^ab^	2140.38 ± 208.37^abc^	2059.33 ± 278.87^bc^	2386.50 ± 99.76^a^	1976.71 ± 472.75^c^
	chroma	19.00 ± 2.07^a^	13.56 ± 1.06^b^	14.78 ± 4.32^b^	17.04 ± 3.82^ab^	15.93 ± 3.36^ab^
	Hue	0.76 ± 0.04^a^	0.82 ± 0.04^a^	0.83 ± 0.07^a^	0.82 ± 0.11^a^	0.78 ± 0.07^a^
CS	Alcohol content (v/v, %)	15.13 ± 0.13^ab^	14.9 ± 0.49^ab^	14.38 ± 1.18^b^	15.04 ± 0.59^ab^	15.68 ± 0.38^a^
	Residual sugar (g/L)	3.73 ± 0.98^b^	3.98 ± 0.60^ab^	4.35 ± 0.69^ab^	4.51 ± 0.56^ab^	4.86 ± 0.39^a^
	Titratable acidity (g/L, Tartaric acid)	6.30 ± 0.62^ab^	5.95 ± 0.78^ab^	5.18 ± 0.75^b^	6.44 ± 0.72^a^	5.50 ± 1.09^ab^
	Volatile acid (g/L)	0.55 ± 0.04^a^	0.42 ± 0.07^a^	0.51 ± 0.06^a^	0.49 ± 0.11^a^	0.51 ± 0.12^a^
	Free SO2	28.50 ± 5.02^c^	35.7 ± 4.60^ab^	30.50 ± 3.91^bc^	36.38 ± 2.23^a^	32.80 ± 2.40^abc^
	pH	3.49 ± 0.25^b^	3.61 ± 0.18^ab^	3.84 ± 0.10^a^	3.76 ± 0.32^ab^	3.73 ± 0.20^ab^
	Total anthocyanins(mg/L)	351.38 ± 78.65^a^	374. ± 128.91^a^	377.08 ± 78.05^a^	410.81 ± 134.48^a^	444.10 ± 102.43^a^
	Total tannin(g/L)	3.70 ± 1.50^a^	5.50 ± 0.99^a^	4.49 ± 1.62^a^	3.90 ± 1.12^a^	4.58 ± 0.92^a^
	Total phenols(mg/L)	2401.38 ± 91.44^a^	2140 ± 208.37a^b^	2010.17 ± 316.40^b^	2387.19 ± 111.23^a^	1772.70 ± 408.90^b^
	chroma	18.47 ± 1.20^a^	13.5 ± 1.06^b^	12.79 ± 3.47^b^	15.77 ± 3.19^ab^	14.62 ± 2.94^ab^
	Hue	0.76 ± 0.04^a^	0.82 ± 0.04^a^	0.86 ± 0.06^a^	0.84 ± 0.11^a^	0.81 ± 0.06^a^
M	Alcohol content (v/v, %)	15.4 ± 0.2^a^	/	15.57 ± 0.98^a^	15.85 ± 0.35^a^	15.85 ± 0.15^a^
	Residual sugar (g/L)	4.55 ± 0.55^a^	/	4.5 ± 0.71^a^	5.15 ± 0.25^a^	4.55 ± 0.55^a^
	Titratable acidity (g/L, Tartaric acid)	5.80 ± 0.40^ab^	/	5.63 ± 0.50^ab^	6.45 ± 0.05^a^	4.80 ± 0.30^b^
	Volatile acid (g/L)	0.55 ± 0.04^a^	/	0.56 ± 0.10^a^	0.57 ± 0.01^a^	0.43 ± 0.11^a^
	Free SO2	31.50 ± 1.50^a^	/	31.00 ± 5.10^a^	34.50 ± 1.50^a^	35.00 ± 0.00^a^
	pH	3.69 ± 0.01^a^	/	3.78 ± 0.15^a^	3.84 ± 0.04^a^	3.78 ± 0.09^a^
	Total anthocyanins(mg/L)	392.25 ± 16.25^a^	/	469.50 ± 75.98^a^	515.50 ± 16.50^a^	362.25 ± 0.25^a^
	Total tannin(g/L)	2.83 ± 0.71^a^	/	3.25 ± 0.54^a^	4.76 ± 0.32^a^	3.49 ± 0.72^a^
	Total phenols(mg/L)	2165.00 ± 68.00^b^	/	2157.67 ± 136.31^b^	2383.75 ± 68.25^ab^	2486.75 ± 0.25^a^
	chroma	20.07 ± 2.89^a^	/	18.74 ± 2.88^a^	22.11 ± 2.44^a^	19.19 ± 0.76^a^
	Hue	0.76 ± 0.01^a^	/	0.76 ± 0.03^a^	0.74 ± 0.03^a^	0.72 ± 0.03^a^

*Note*: N-S represents the order of regions from left to right, arranged from north to south. Different Latin letters indicate significant differences according to the Tukey HSD test (*P* < 0.05).

#### 3.3.2 Classification of wines by geographical origin based on volatile compounds

Specific volatile compounds are important markers that can reflect the terroir effects of regions. There is evidence to support the implementation of traceability for wines from different subregions. This study conducted dimensionality reduction analysis on 65 quantified volatile compounds.

**Fig. 4:**
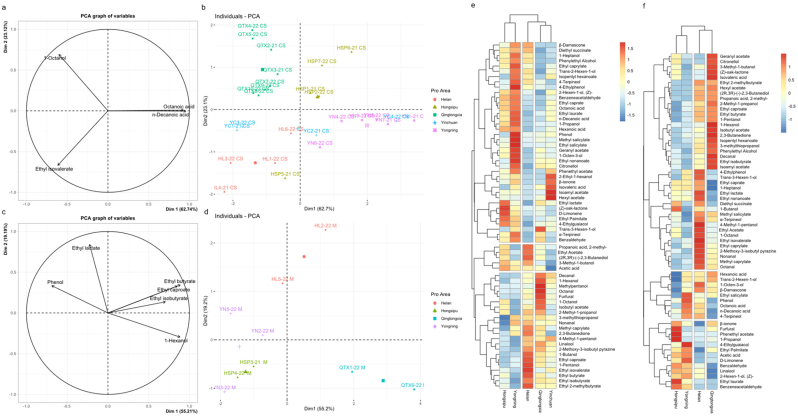
**Factor maps (a & c), individual factor maps (b & d), and heatmap (e & f) of CS (*P* < 0.01) and M (*P* < 0.01) wine volatile compounds in EFHM.** The horizontal axis represents the wine region; vertical axis, the oenological parameter; and blue to red, the content from low to high of e & f.

First, the PCA method was employed to analyze the correlation between CS and M wines from the different subregions of EFHM and key volatile compounds, as shown in Fig. 4. The contribution rates of CS wine PC1 and PC2 are 62.7 % and 23.1 %, whereas those of M wine PC1 and PC2 are 55.2 % and 19.2 %, respectively. Compounds such as ethyl isobutyrate and n-decanoic acid in the variable plot were the main vectors with significant differences obtained through significance screening in the CS wine (Fig. 4a), whereas hexanol and ethyl butyl were the main vectors with significant differences in the M wine (Fig. 4c). The distribution of wines from different subregions was achieved at different coordinates through the correlation between volatile compounds. The distribution of CS wines from different subregions is shown in Fig. 4b. The HSP wines located in the first quadrant, aligned with the positive coordinate direction of PC1 and PC2, and positively correlated with octanoic acid, n-decanoic acid, and 1-octanol has aroma such as floral and fruity aromas. The QTX region was located in the second quadrant, aligned with the positive coordinate direction of PC2, and positively correlated with 1-octanol, which has aromas such as lemon, citrus, and jasmine. HL wines were located in the third quadrant, in the negative coordinate direction of PC1 and PC2, and were negatively correlated with octanoic acid, n-decanoic acid, and 1-octanol and positively correlated with ethyl isovalerate with fruity aroma. The YN wines were located in the fourth quadrant, in the positive coordinate direction of PC1 and the negative coordinate direction of PC2. They were positively correlated with octanoic acid, n-decanoic acid, and ethyl isovalerate and negatively correlated with 1-octanol. The YC wines were in the negative coordinate direction of PC2 and were positively correlated with ethyl isovalerate. The distribution of M wines from different subregions is illustrated in Fig. d. The HL wines were located on the positive axis of PC2, positively correlated with ethyl lactate with a milky flavor, and negatively correlated with ethyl isobutyl, ethyl butyl, and ethyl caproate. The YN wines were located on the negative half-axis of PC2 and were positively correlated with ethyl lactate and phenol. The HSP wines were located in the third quadrant and were negatively correlated with several key volatile compounds. The QTX wines were located in the fourth quadrant, consistent with the positive coordinate direction of PC1, and were positively correlated with 1-hexanol, ethyl isobutyl, ethyl butyl, and ethyl caproate, particularly with 1-hexanol, which has a grassy aroma. They were negatively correlated with phenol and ethyl lactate, consistent with the prominent green flavor in sensory evaluation.

To better understand the similarities and differences between the CS and M wines from the different subregions of EFHM, volatile compounds were used as variables for cluster analysis to explore the distance of the terroir relationships among wines from different subregions. The volatile compound profiles of the CS and M wines are illustrated in Fig. 4e & 4f. According to the similarity of volatile compounds, the parameters were divided into three groups from top to bottom, and the subregions were clustered into three from right to left. The grouping and ranking of the CS and M wines based on their geographical location determined by volatile compounds were roughly the same. Among them, the YC and QTX regions were clustered together in the shortest hierarchical distance owing to similar volatile compound profiles (the M wine was clustered separately). The HL region was separately clustered within the middle layer distance. The YN and HSP regions were clustered together owing to their similar volatile compound profiles and located at the farthest hierarchical distance from others. The similarity between the CS samples from the YC and QTX regions with similar contours is that the content of the first two groups of compounds was generally lower, whereas the content of the third group of compounds, such as decanal, was generally relatively higher. The difference is that some substances, such as 2-ethyl-1-hexanol, had higher levels in the YC region, whereas other substances, such as octanal, had higher levels in the QTX region. The difference between the M wines from the QTX region lies in the higher content of the first group of substances, such as geranyl acetate. The difference between the CS wines from the HL region is that the content of the first group of compounds was lower, whereas that of the second and third groups of compounds, such as ethyl acetate, was higher. The difference between the M wines is that the content of the first two groups of compounds, such as 4-ethylphenol, was higher, whereas that of the third group of compounds was lower. The similarity between the YN and HSP regions with similar contours lies in the fact that the content of the first group of compounds, such as benzeneacetaldehyde, was relatively higher in the CS wine, whereas that of the third group of compounds was relatively lower. The difference lies in the higher content of compounds, such as isopentyl hexanoate, in the YN region, whereas compounds such as ethyl lactate had a higher content in the HSP region. The similarity between the two regions in the M wine is that the content of the first group of compounds was lower whereas the content of the third group of compounds, such as d-limonene, was higher. The difference lies in the higher content of 4-ethylguaiacol and other substances in the YN region, whereas the content of furfur and other substances was higher in the HSP region. There were both commonalities and individual characteristics among the regions and varieties.

To elucidate the specific impact of volatile compounds on wine aroma, the results of HCA and QDA were combined. YN's CS and M wines with the best sensory evaluation results, the content of 4-terpineol, phenol, cis-2-hexen-1-ol, ethyl salicylate, octanoic acid, and n-decanoic acid that with fruity, floral, green, and cheesy flavors were higher than that of other subregions, suggesting that they may be key compounds affecting the core aroma of wines from EFHM. The formation of the first three substances (variety aroma) mainly came from the accumulation and metabolism of fruit precursors and the release of glycosides(Parker et al., 2012), with profound terroir imprints. The floral aroma of wines from the YN and HSP regions was more prominent. The cluster analysis revealed that ethyl lactate, which has a floral aroma, was present in higher levels in wines from these two regions and may be one of the key compounds affecting the floral aroma in the regions. The CS wines from the QTX and YN regions have a relatively prominent green flavor. The cluster analysis revealed that volatile phenols (4-ethylguaiacol), C6 alcohols (cis-2-exen-1-ol, 1-octen-3-ol), higher alcohols (1-hetanol), and esters ((*Z*)-aok-lactone) with green flavor were present in these two regions and may be key compounds affecting the green flavor of the regions. The mushroom flavor of the YN and HL wine regions was relatively prominent, and 1-octen-3-ol, which has a mushroom flavor, may be one of the key substances affecting the mushroom flavor of wine in these regions.

#### 3.3.3 Classification of wines by geographical origin based on phenolic compounds

Specific phenolics are important markers that can reflect the terroir effects of regions. There is evidence to support the traceability of wines from different subregions. In this study, dimensionality reduction analysis was conducted on 16 quantified monomeric phenols.

**Fig. 5:**
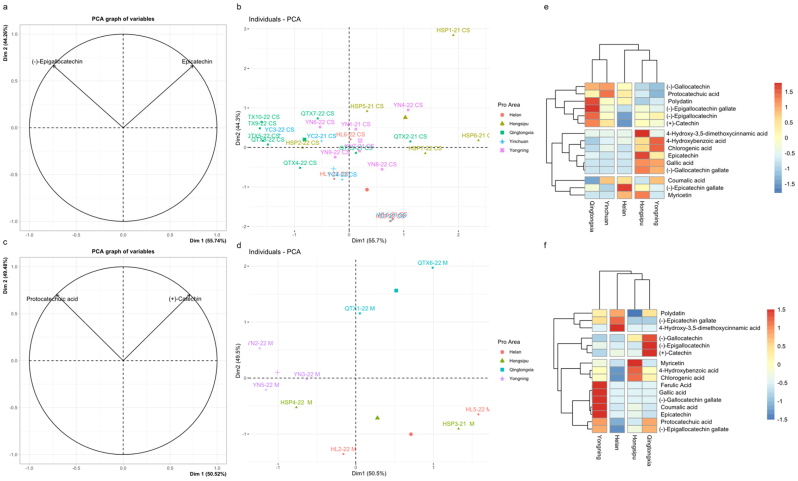
**Factor maps (a & c, *P* < 0.001), individual factor maps (b & d, *P* < 0.001), and heatmap (e & f) of CS (*P* < 0.01) and M (*P* < 0.01) wine phenolic compounds in EFHM.** The horizontal axis represents the wine region; vertical axis, the oenological parameter; and blue to red, the content from low to high of e & f.

First, the correlation between the CS and M wines from different subregions and phenols was analyzed via PCA. The contribution rates of CS wine PC1 and PC2 were 55.7 % and 44.3 %, whereas the contribution rates of M wine PC1 and PC2 were 50.5 % and 49.5 %, respectively. The compounds EGC and EC in the variable plot were the main vectors with significant differences obtained through significance screening in CS wine (Fig. 5a), whereas the compounds protocatechuic acid (PA) and C were the main vectors with significant differences in the M wine (Fig. 5c). The differences in the coordinate distribution of wine samples from different subregions were due to their varying correlations with these compounds. The distribution of CS wines from different subregions is shown in Fig. 5b. The HSP wines were mostly concentrated in the first quadrant and positively correlated with EC. The wines from the YN region were mostly concentrated in the positive coordinate direction of PC1 and positively correlated with EC and EGC, whereas the wine samples from the QTX region were mostly concentrated in the second quadrant and positively correlated with EGC. The wines from the HL regions were mostly concentrated in the fourth quadrant and negatively correlated with EGC. The distribution of M wines from different subregions is illustrated in Fig. 5d. The QTX wines were concentrated in the first quadrant and positively correlated with C, whereas the wines from the YN region were in opposition to PC1, positively correlated with PA, and negatively correlated with C. The wines from the HSP and HL regions were in opposition to PC2 and were negatively correlated with PA and C. The QTX wines were concentrated in the first quadrant and positively correlated with C, whereas the YN wines were in opposition to PC1, positively correlated with PA, and negatively correlated with C.

To better understand the similarities and differences between the CS and M wines from the different subregions of EFHM, 16 monomeric phenols were used as variables for cluster analysis to explore the distance of the terroir relationships among wines from the different subregions. The phenol profiles of the CS and M wines are illustrated in Fig. 5e & 5f. According to the similarity of phenols, the parameters were divided into three groups from top to bottom, and the subregions were clustered into three from right to left. The phenol profile of the CS wine is shown in Fig. 5e. The YN and HSP regions were clustered together within the shortest hierarchical distance owing to their similar contours. The similarity between the two production areas lies in the higher content of second-group substances, such as hydroxycinnamic acid (4HA, CGA, GA, etc.) and flavanols (EC, GCG), particularly in the HSP region. This may be due to the lower temperature in the hottest month (July), which was conducive to the synthesis of these compounds in the region (Martínez-Gil et al., 2018). The difference is that SA, EC, and myricetin had the highest content in the HSP production area, whereas 4-HA and CGA had the highest content in the YN production area. The HL region is clustered separately, with the difference being the higher content of ECG and myricetin in the third group of compounds. Compared to others, YC and QTX regions are the farthest clusters. The similarity between the two production areas is that the content of the first group of substances, such as C and GC, was higher, whereas the content of the second and third groups of substances was lower, which resulted in their accumulation within a shorter hierarchical distance. The difference is that PA and CMA had the highest content in the YC region, whereas polydatin, EGCG, EGC, and C had the highest content in the QTX region. The phenol profile of the M wine is shown in Fig. 5f. The region arrangement of the M wine is better than that of CS, showing a significant geographical arrangement and terroir effect. HSP and QTX, two adjacent regions located in the south, have similar phenolic profiles and were clustered within the shortest hierarchical distance. The similarity between the two regions is the lower content of the first group of substances. The difference lies in the high contents of myricetin, 4HA, and CGA in the M wine from the HSP region, whereas GC, EGC, and C had high contents in the M wine from the QTX region. Next, from near to far, the HL region in the north and the YN region in the middle were separately clustered. The contents of polydatin, ECG, and SA in the first group of monomeric phenolic compounds were the highest in the HL production area. The contents of the third group of monomeric phenolic compounds, such as fatty acid, gallic acid, and coumaric acid, were the highest in the YN region. The CS and M wines from the HL region were separately clustered possibly due to the generally low content of most monomeric phenols. Supplementary Table S1 and Fig. S1 show that the sunshine hours in this production area are relatively low and that the temperature in the hottest month is high, which negatively affects the accumulation of phenolic substances.

### 3.4 Correlation between sensory scores and flavor compounds in EFHM wine

Correlation analysis is the analysis of two or more correlated variable elements to measure the degree of their correlation. The overall wine aroma is not simply the sum of various aroma compounds but is ultimately formed through complex interactions, such as addition, synergy, and masking. In this study, a correlation analysis on data, including sensory evaluation indicators, basic physicochemical indicators, and volatile compounds, was conducted to better understand the relationship between flavor compounds and sensory attributes as well as their inherent connections and to better elucidate the main compounds that affect the sensory attributes of wine from the eastern foothills of Helan Mountain. First, to more clearly observe the results, indicators with absolute values greater than 0.8 were selected from various factors and retained for recorrelation analysis. As shown in Supplementary Fig. S4, there is a significant correlation between sensory characteristics and volatile compounds. The coordination and elegance of aroma were significantly positively correlated (*r* ≥ 0.5) with cis-2-exen-1-ol (C6 compound content) with green flavor, ethyl palmitate (other ester contents) with fruity and floral aromas, n-decanoic acid, and octanoic acid with cheesy and fatty flavors. These compounds are key indicators of the coordination and elegance of wine aroma. C6 compounds, including cis-2-exen-1-ol, were also significantly positively correlated with aroma richness (*r* ≥ 0.5). These substances were key compounds that determine the concentration of green flavor in wine. In addition, a significant correlation was observed between flavor compounds. There was also a significant positive correlation between the content of higher alcohols, fatty acids, ethyl esters, and acetic esters, the correlation coefficient between the highest content of higher alcohols (see Supplementary Fig. S4) and the total amount of volatile compounds was 1. A significant positive correlation was also observed between several substances, such as cis-2-hexen-1-ol (C6 compound), n-decanoic acid, octanoic acid (fatty acid), ethyl laurate, and ethyl palmitate (other esters). Moreover, there was a significant positive correlation between esters and alcohols (ethyl caproate, ethyl butyl, and 1-pentanol). A significant negative correlation between esters (ethyl 2-methylbutyl and ethyl acetate). Both ethyl isovalerate and ethyl 2-methylbutyrate were negatively correlated with pH. The correlation between these compounds may have stemmed from the same or related synchronous metabolic pathway, such as the production of higher alcohols and acetate esters originating from the amino acid metabolism(Saerens, Delvaux, Verstrepen, & Thevelein, 2010), the production of fatty acids and their esters originates from the metabolism of coenzyme A thioesters, whereas fatty acids and higher alcohols were substrates for the synthesis of ethyl esters (Dennis et al., 2012; Saerens et al., 2006). Or it may stem from the conversion between substances (fatty acids → C6 aldehydes → C6 alcohols → esters → ester exchanges).

## 4 Conclusion and prospect

In summary, the richness and coordination of fruity, floral, green, spice, and dried fruity flavors are key indicators of the flavor characteristics of EFHM wine. Compounds such as cis-2-exen-1-ol, ethyl palmate, octanoic acid, and n-decanoic acid are key compounds that determine the core aroma, coordination, and elegance of EFHM wine. Cis-2-exen-1-ol is a key compound that determines the intensity of the green flavor in the EFHM wine. Ethyl salicylate and ethyl laurate are key compounds that affect the floral aroma of EFHM wine. Furthermore, 1-octen-3-ol is a key compound that affects the mushroom flavor of wine. The sensory and chemical properties of wines from different subregions have varying characteristics. The CS wine from the HSP region has a prominent floral aroma and a high content of ethyl laurate with floral notes. CS wines from the QTX region have prominent green and spice flavors, with high levels of 4-ethylguaiacol (volatile phenol), cis-2-exen-1-ol, 1-octen-3-ol (C6 alcohol), 1-hetanol (higher alcohol), (*Z*)-aok-lactone (ester), and flavanols. The five core aromas of YN region wines were prominent and balanced. The contents of C6 compounds, other esters, volatile phenols, and phenolic acids were relatively high. The CS wine from the YC region has a prominent fruity aroma and a high content of other esters. The CS wine from the HL region has prominent green, fruity, and spice flavors. The contents of ethyl ester and C6 compounds were relatively high. From the perspective of sensory scores, wines produced under lower accumulated temperatures, longer sunshine hours, and sandy or yellow loam soil have better quality. In geographical traceability based on chemical parameters, that chromaticity, total phenols, ethyl isobutyrate, n-decanoic acid, (−)-epigallocatechin, and epigallocatechin were key indicators for identifying the region of CS wines, whereas total acids, total phenols, hexanol, ethyl butyrate, protocatechuic acid, and (+)-catenin were key indicators for achieving region discrimination of M wines. The cluster analysis revealed that the winemaking areas that are geographically close have more similar chemical profiles.

In the future, we can continue to collect young wines from multiple vintages for validation and conduct targeted research to confirm the regulation of the accumulation of flavor compounds in wine through the improvement of related terroir composite factors. This will enable targeted improvements in the quality of wine, resulting in the production of wines with typical styles, excellent quality, and diverse categories. This includes improving nitrogen metabolism, increasing the accumulation of aroma precursors, and regulating the synthesis of volatile compounds, such as isoprene, pyrazine, C6 compounds, esters, and phenols, through various aspects, such as appropriate fertilization, spray induction, deficit irrigation, light regulation, and optimization of fermentation processes.

## CRediT authorship contribution statement

**Xue Zhang:** Writing – review & editing, Writing – original draft, Supervision, Formal analysis, Data curation, Conceptualization. **Hui Yang:** Writing – review & editing, Writing – original draft, Supervision, Formal analysis, Conceptualization. **Na Liu:** Visualization, Supervision, Resources, Investigation. **Jian Sun:** Visualization, Supervision, Resources, Investigation. **Ruijia Yao:** Validation, Formal analysis, Data curation. **Fangzhou Shi:** Validation, Formal analysis, Data curation. **Jiming Li:** Resources, Project administration. **Wenguang Jiang:** Resources, Project administration. **Hongying Li:** Resources. **Qingchen Zhang:** Writing – review & editing. **JunXiang Zhang:** Writing – review & editing, Supervision, Funding acquisition, Conceptualization.

## Declaration of competing interest

The authors declare that they have no known competing financial interests or personal relationships that could have appeared to influence the work reported in this paper.

## Data Availability

Data will be made available on request.

## Appendix A Supplementary data

Supplementary material 1 Image 1

Supplementary material 2 Image 2

Supplementary material 3 Image 3

Supplementary material 4 Image 4

Supplementary material 5 Image 5
